# Multi-scale Visualization of Molecular Architecture Using Real-Time Ambient Occlusion in Sculptor

**DOI:** 10.1371/journal.pcbi.1004516

**Published:** 2015-10-27

**Authors:** Manuel Wahle, Willy Wriggers

**Affiliations:** 1 School of Biomedical Informatics, University of Texas Health Science Center at Houston, Houston, Texas, United States of America; 2 Department of Mechanical and Aerospace Engineering, Old Dominion University, Norfolk, Virginia, United States of America; The Scripps Research Institute, UNITED STATES

## Abstract

The modeling of large biomolecular assemblies relies on an efficient rendering of their hierarchical architecture across a wide range of spatial level of detail. We describe a paradigm shift currently under way in computer graphics towards the use of more realistic global illumination models, and we apply the so-called ambient occlusion approach to our open-source multi-scale modeling program, Sculptor. While there are many other higher quality global illumination approaches going all the way up to full GPU-accelerated ray tracing, they do not provide size-specificity of the features they shade. Ambient occlusion is an aspect of global lighting that offers great visual benefits and powerful user customization. By estimating how other molecular shape features affect the reception of light at some surface point, it effectively simulates indirect shadowing. This effect occurs between molecular surfaces that are close to each other, or in pockets such as protein or ligand binding sites. By adding ambient occlusion, large macromolecular systems look much more natural, and the perception of characteristic surface features is strongly enhanced. In this work, we present a real-time implementation of screen space ambient occlusion that delivers realistic cues about tunable spatial scale characteristics of macromolecular architecture. Heretofore, the visualization of large biomolecular systems, comprising e.g. hundreds of thousands of atoms or Mega-Dalton size electron microscopy maps, did not take into account the length scales of interest or the spatial resolution of the data. Our approach has been uniquely customized with shading that is tuned for pockets and cavities of a user-defined size, making it useful for visualizing molecular features at multiple scales of interest. This is a feature that none of the conventional ambient occlusion approaches provide. Actual Sculptor screen shots illustrate how our implementation supports the size-dependent rendering of molecular surface features.

## Author Summary

In this work, we present an implementation of screen space ambient occlusion (SSAO), which supports the visualization and modeling of multi-scale biophysical data, such as atomic structures and 3D density maps, at multiple scales of interest. The ever-growing size of macromolecular assemblies presents a formidable challenge to molecular modeling programs. Ambient occlusion (AO) has recently received a lot of attention in high-quality rendering engines as well as in video games. The technique provides an acceptable real-time approximation of global illumination, by adding realistic cues about the indirect shading of the surface topology. SSAO leverages the computational power and flexibility of graphical processors, which are present on almost all modern video cards. By using SSAO, we put an emphasis on compatibility and speed, while delivering the visual benefits of AO at a user-selected level of detail. Our work is particularly timely because many molecular graphics packages are currently adopting global illumination schemes, but our work is unique in providing size-specificity of the shaded features.

This is a *PLOS Computational Biology* Software paper

## Introduction

Multi-scale molecular modeling is concerned with the computational integration and interpretation of spatio-temporal biophysical data from various experimental origins [1–3]. During the last decade, we have developed computational techniques for interpreting small-angle X-ray scattering (SAXS) and electron microscopy (EM) data [4], the modeling of structural flexibility [5], and visualization of functional “machine parts” using computer graphics [6, 7]. The unifying goal of these efforts was to observe and to account for functional architecture and dynamics in native environments (solution or vitreous ice) or *in silico* and then to reconstruct and interpret the 3D shapes of large biomolecular assemblies across multiple spatial or time scales. Ideally, such large structures are visualized at the atomic level; however, volumetric 3D maps have become increasingly common [8].

Early multi-resolution modeling was carried out manually [9], based on the visual representations displayed on a computer screen, although algorithmic approaches soon emerged [10]. Despite the algorithmic advancements, it is essential that the graphical depictions are of high visual quality to validate the precision or accuracy of models, to help the user perceive the spatial architecture, and to facilitate scientific communication. Computer graphics have been used for the visualization of molecules since the rise of graphics workstations [11, 12]. Availability and quality of rendering were mainly driven by the development of more advanced hardware. Although special purpose graphics hardware was available since the early 1980s, it was not until the early 1990s that 3D graphics rendering, actively performed by a processor on a graphics card, became increasingly prevalent in mass-market personal computers. When OpenGL emerged as a common interface for 3D graphics programming, it eventually resulted in a widespread support of hardware acceleration available in commodity computers.

Mostly driven by the gaming industry, mass market GPUs steadily improved in terms of processing power and flexibility. During the early 2000s, programmable shading capabilities were added to these dedicated rendering processors [13, 14]. Up to then, geometry was projected onto the screen through a fixed functionality pipeline. A certain set of parameters like color and light positions could be adjusted, but no further customizations were possible without considerate efforts or workarounds. Although still being mainly targeted towards graphics processing, GPUs nowadays are even used routinely for general purpose scientific computing.

**Fig 1:**
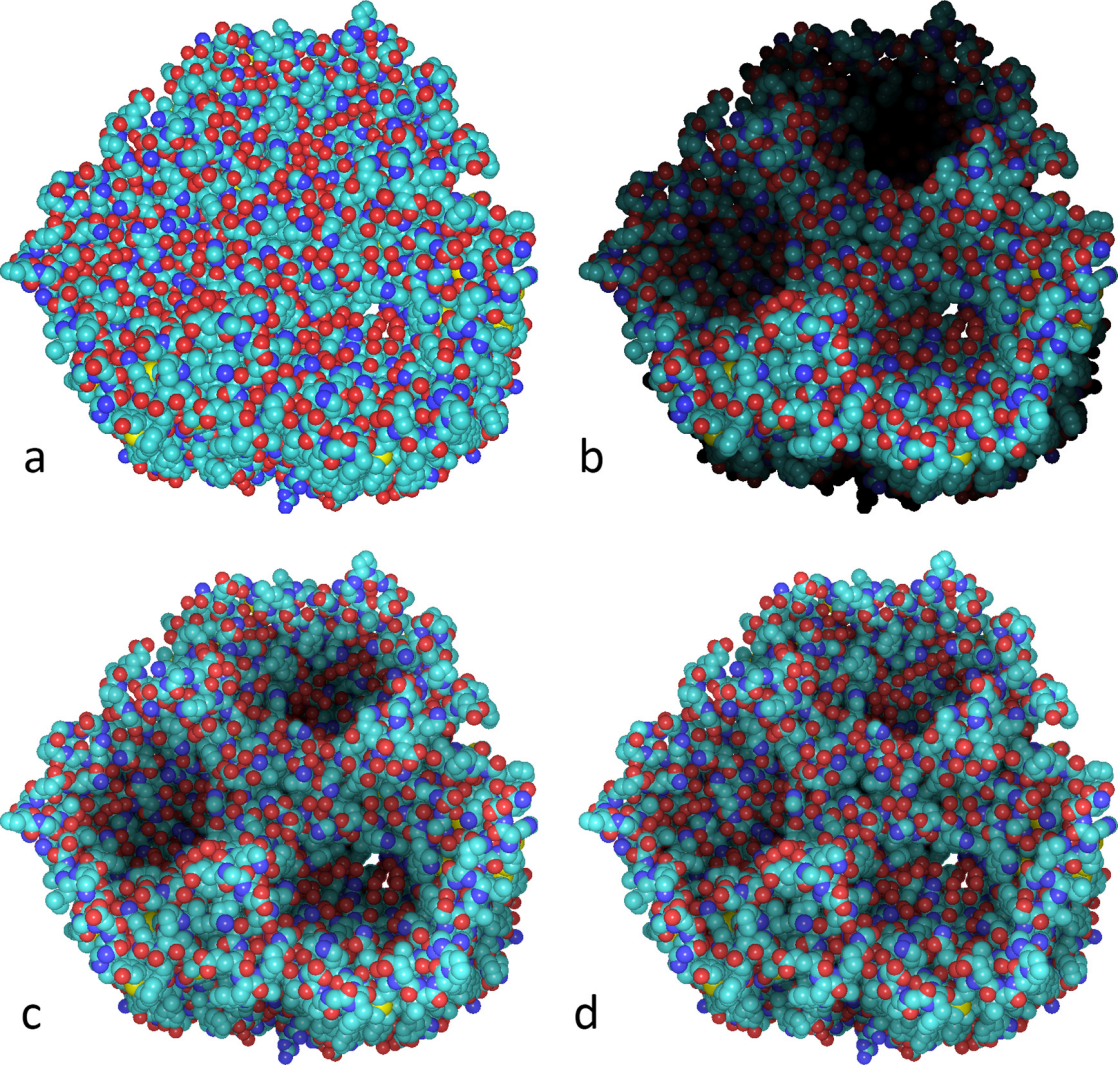
Local and SSAO rendering of an atomic structure of a maltoporin protein (PDB ID 1AF6). (a) Standard local lighting, which provides only a locally acceptable approximation of real world lighting. (b) Depth cueing, which unevenly shades the three (slightly tilted) channels and looks unrealistic at the bottom. (c-d) SSAO, at two different sampling sizes that emphasize spatial features according to user preference. The protein is shown in van der Waals mode in an orientation corresponding to Fig 2 in [16]. All molecular graphics figures in this paper were created with Sculptor version 2.1 [17, 18].

At the time of the founding of PLoS Computational Biology, in the mid 2000s, interactive graphics software relied on strongly simplified approximations of real-world lighting such as Phong or Gouraud shading (Fig 1a) and depth cueing (Fig 1b), which do not deliver convincing cues about the spatial characteristics of represented structures [15]. This was due to the limitation that lighting could be computed only locally at each point in real time. In depth cueing, the color of objects was blended with that of the background to create a distance-dependent “fog.” This approach was unrelated to lighting and did not look convincing for large structures because the “fog bank” changed direction with the viewpoint (Fig 1b) and the effect was uniform at a given pixel depth. A more realistic rendering of shadows and ambient light in real time seemed out of reach: Such *global* lighting effects, where objects in the scene affect the lighting of other objects (Fig 1c and 1d), used to require an expensive ray tracing that had to be computed off-line in batch mode [15].

**Fig 2:**
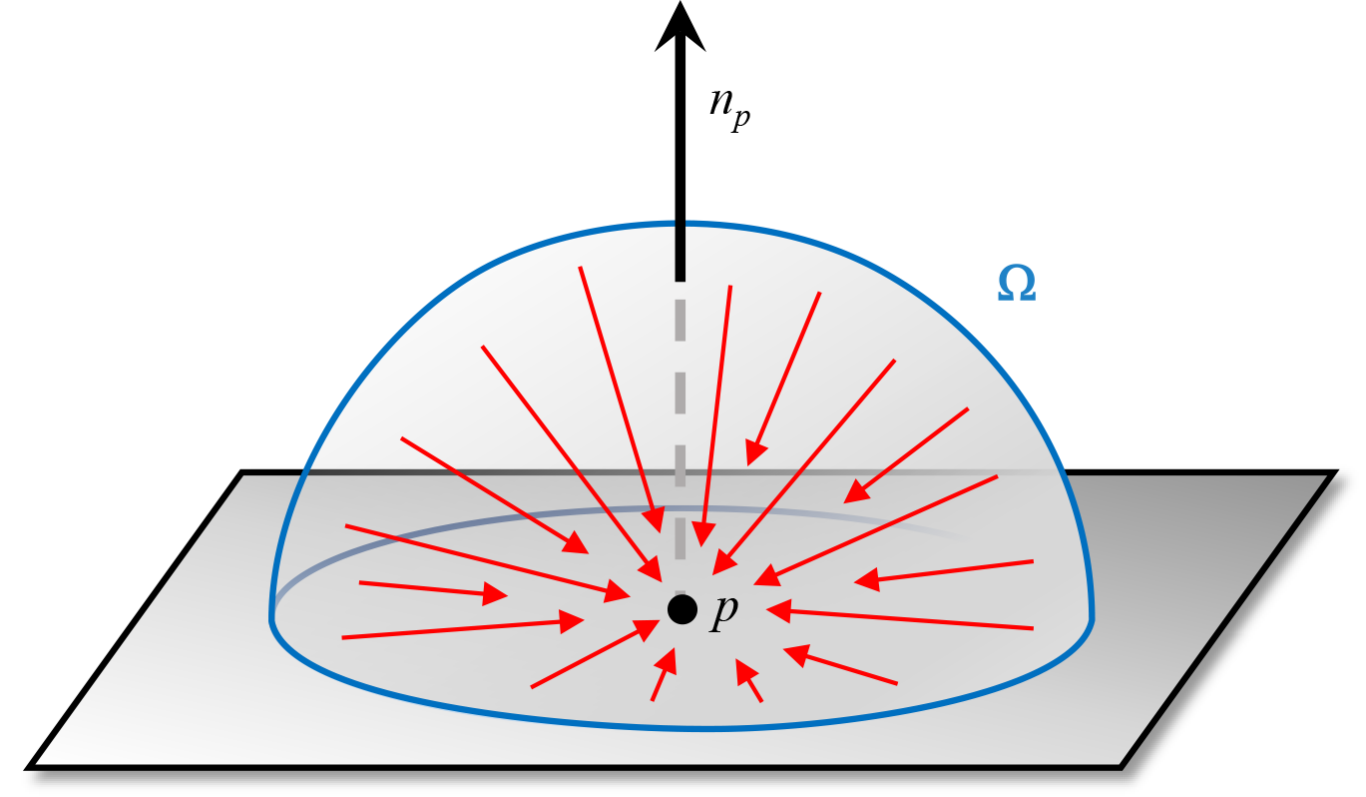
Global illumination by ambient light rays (red) emanating from a hemisphere Ω. The illumination is governed by a weighted sum of unblocked light rays (red) that reach the surface point *p*, where the weights are given by the cosine of the incident angle relative to the surface normal *n*_*p*_. [16, 21, 22].

In 2007, however, Vladimir Kajalin invented screen space ambient occlusion (SSAO) while working at the German video game company Crytek on the PC game Crysis [19]. It was already known that computer renderings looked more realistic when an ambient background illumination was considered [20]. Ambient occlusion (AO) describes how much of the ambient light (emanating from a uniform lighted sky) is blocked by the scene geometry from reaching a surface point (Fig 2). The occlusion can be computed by integrating the visibility (blockage) function over the hemisphere with respect to the projected solid angle [21, 22]. Typically the calculation requires an expensive pre-processing step, but SSAO uses pixel depth to form an AO map (see Design and Implementation), which opened the door to inexpensive post-processing [23]. SSAO thus enabled AO for real-time applications such as computer games and molecular graphics.

**Fig 3:**
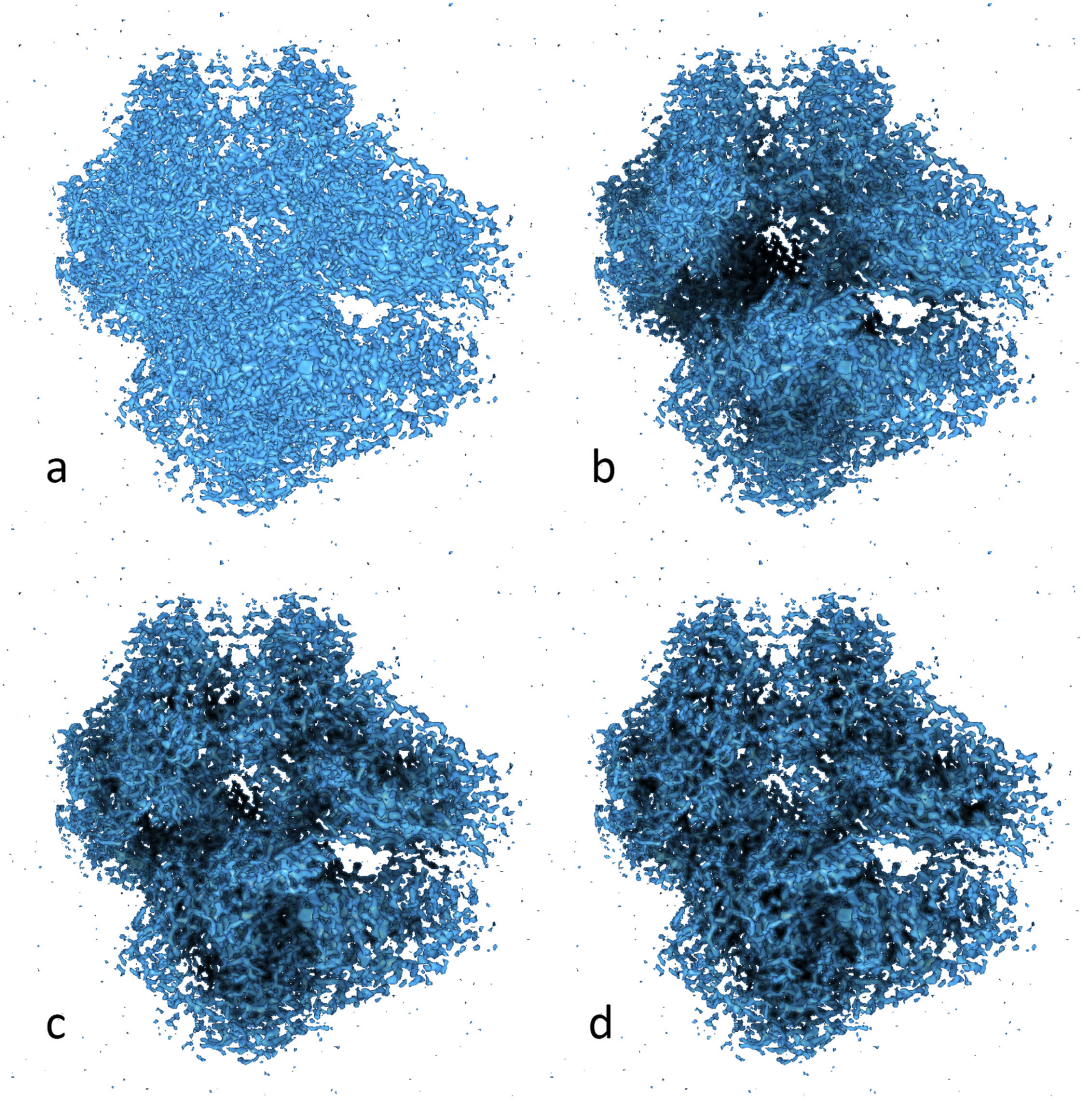
Volumetric representation of a 2.2 Å resolution cryo electron microscopy density map of beta-galactosidase in complex with a cell-permeant inhibitor (EMDB ID 2984). (a) Standard local lighting. (b-d) SSAO, at three different sampling sizes that emphasize tertiary (b), secondary (c), and primary (d) spatial features according to user preference.

AO is observed when two surfaces are close. As long as the effect is not surpassed by strong shadows or direct lighting, a highly diffuse darkening can be observed between the surfaces. Good examples are creases or pockets, or any kind of deeper concavity in objects. Applying this effect to space-filling representations of biomolecules, or to isosurfaces of volumetric maps, greatly enhances the perception of their spatio-structural characteristics. Fig 1 shows the effect applied to the atomic structure of a protein structure; in Fig 3 it is applied to a volumetric map. Both figures show how AO contributes to the perception of spatial structure at a user-defined level of detail.

This paper presents an adaptation of SSAO to real-time modeling and visualization of multi-resolution biomolecular structures. To handle the additional computational load, our implementation takes advantage of modern graphics processing units (GPUs). The method is implemented in our open source modeling software, Sculptor [6, 17], which is freely available at http://sculptor.biomachina.org. In Results, we will describe how SSAO is particularly beneficial for multi-scale docking and registration studies that require highlighting of specific size cavities and pockets through improved lighting, for which Sculptor was designed. In Availability and Future Directions we will also compare our implementation to ongoing developments in conventional molecular graphics programs.

## Design and Implementation

### Related Work

Among the many techniques for computing global lighting aspects, AO is one of the most popular. The main idea of AO is to calculate an occlusion factor *A*_*p*_ for ambient light at each point *p* in the scene, which approximates the light distribution (Fig 2). *A*_*p*_ results from the integral over the visible hemisphere Ω over the blocked incoming light energy *ρ*(*L*) based on the distance to the nearest obstacle *L*(*p*, *u*) in direction *u* [16]: *A*_*p*_ = ∫_Ω_ *ρ*(*L*(*p*, *u*)) *n*_*p*_ ⋅ *u* *du*. *A*_*p*_ thus accounts for the loss of ambient light by blocking of incident rays by nearby scene geometry. A variety of techniques are used to approximate AO in practice. One approach is to cast multiple rays from the surface point and testing for intersection of the rays with surrounding surfaces [24]. In [25] the AO is computed by approximating geometry with disks to compute a per vertex value. In [26] precomputed radiance transfer terms are used to efficiently shade a scene, incorporating an AO effect. In [27] a method for computing AO for molecular visualization is designed. It is, however, restricted to representations that use spheres or cylinders.

All of the above methods aimed to achieve high visual quality at interactive frame rates, but they required either a pre-processing step, or they altered the rendering pipeline, which limited their adoption. When SSAO was introduced in [19], the main innovation was that AO was added to the scene *after* all geometry rendering completed. Due to the post-processing, no additional information or adaption of the rendering pipeline are necessary. This makes SSAO very fast, and it is completely independent from the scene complexity. In [28] an early overview of different SSAO techniques is given. More recently, [29] introduced an AO volumes scheme that is comparable to ray tracing in some scenes, [30] introduced a voxel-cone AO that was adopted by NVIDIA (Section 1 in S1 Text), and [16] introduced a fast object-based AO for molecular dynamics rendering.

### Rendering Pipeline

The rendering graphics pipeline is traditionally implemented as follows. The geometry in the scene consists of vertices connected by edges, and every vertex is assigned certain lighting parameters. Vertex positions and those of light sources are used to compute color information. From a given viewpoint (the camera position), the scene is projected onto an image plane, which corresponds to the screen surface. In a process called rasterization, the center of each pixel is associated with information from the projected geometry. Color is the result of interpolating this information from the corresponding original vertices, and depth depends on the distance between camera and original geometry. These per pixel data are stored in so-called fragments.

**Fig 4:**
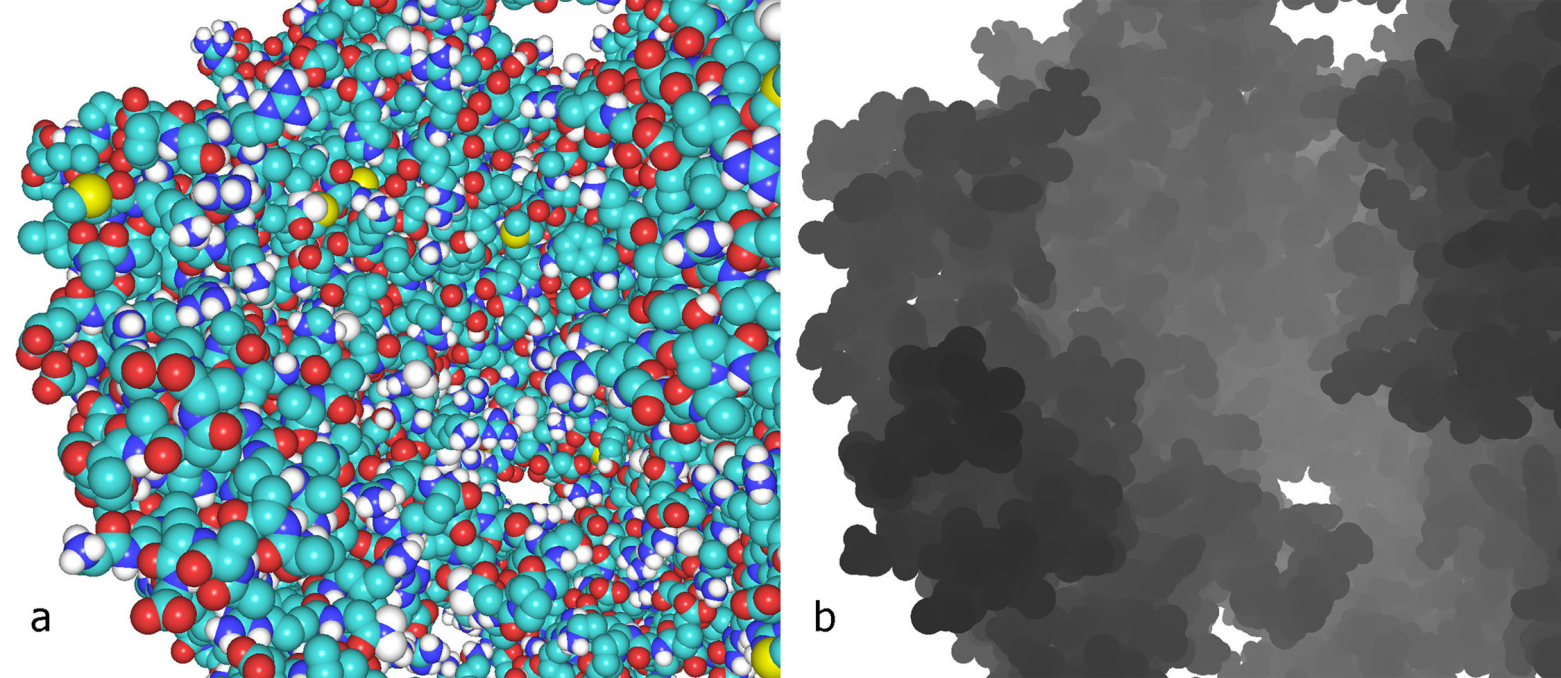
Illustration of buffer contents. (a) Color buffer. (b) Corresponding depth buffer contents in grayscale (black is near and white is far).

**Fig 5:**
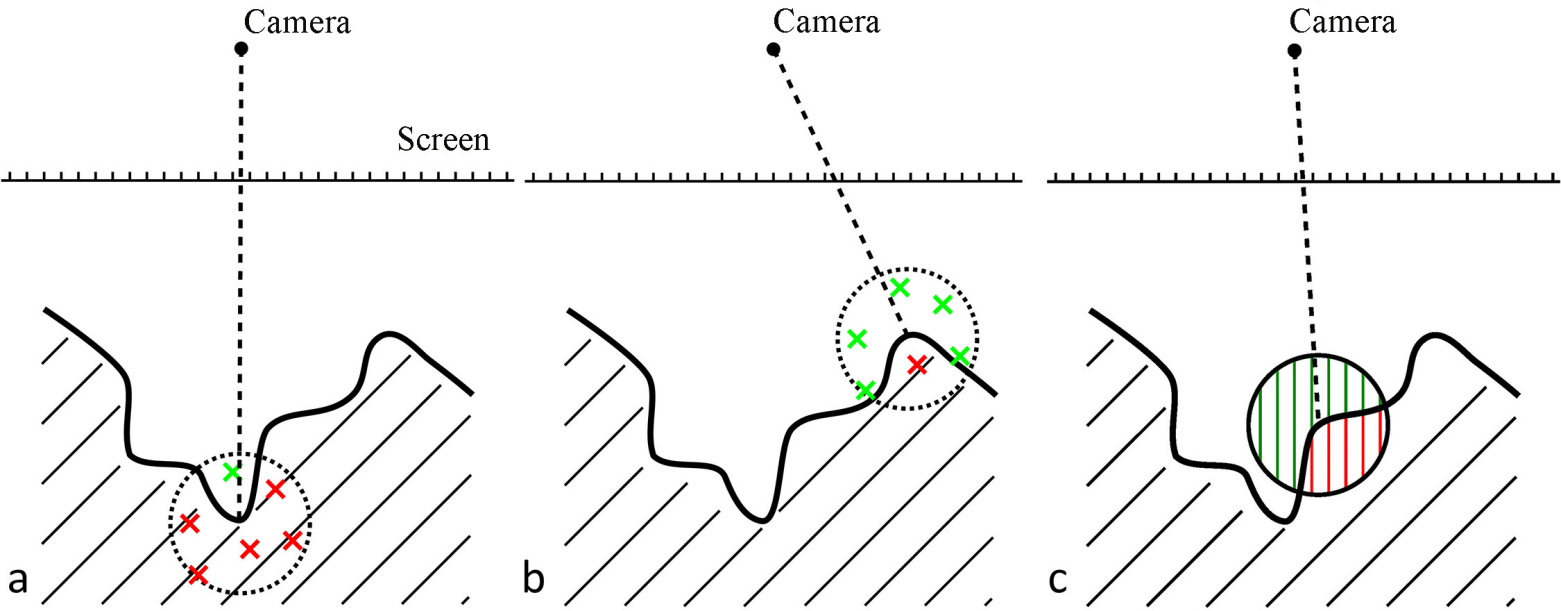
Illustration of SSAO approaches. (a-b) Point-based SSAO for (a) concavity and (b) convexity. Red marks show sample points behind the surface, green ones in front of it. In (a), most points are behind the surface, resulting in high AO. In (b), most points are in front of the surface, resulting in low AO. (c) Line-based SSAO. The green parts of the lines are in front of the surface; the red parts are hidden. Computing the ratio of visible vs. hidden parts yields an AO factor of higher granularity than what can be achieved by the point-based method.

Two different buffers exist: one holding color (displayed on the screen) and the other minimum depth information (Fig 4). Both have the same dimensions as the screen and store data from the fragments. For each newly generated fragment, it must be determined if it will overwrite the previous values in the buffers. In traditional depth cueing (Fig 1b), the pixel color is blended with the background based on the pixel depth, which is unrelated to lighting. In SSAO, the geometry corresponding to every pixel is analyzed to estimate the amount of AO in the original scene. Fig 5 illustrates that at the bottom of a concavity (Fig 5a), AO is high, and so the darkening should be strong. In contrast, at the top of a convexity (Fig 5b), the geometry does not attenuate the ambient lighting. Finally, each pixel’s color is modulated with the AO coefficient.

### Point Sampling

In point sampling [23], sampling is conducted at multiple positions inside a sphere around the current point of interest. From the pixel coordinates and the corresponding depth value, it is possible to compute the 3D position in the scene. This position becomes the center of the sampling sphere (Fig 5a and 5b). Each sampling point is projected onto the image plane, and the sample depth is compared to the depth buffer, yielding the number of visible and hidden sample points. The AO coefficient is then given by the ratio of hidden to visible sample points.

**Fig 6:**
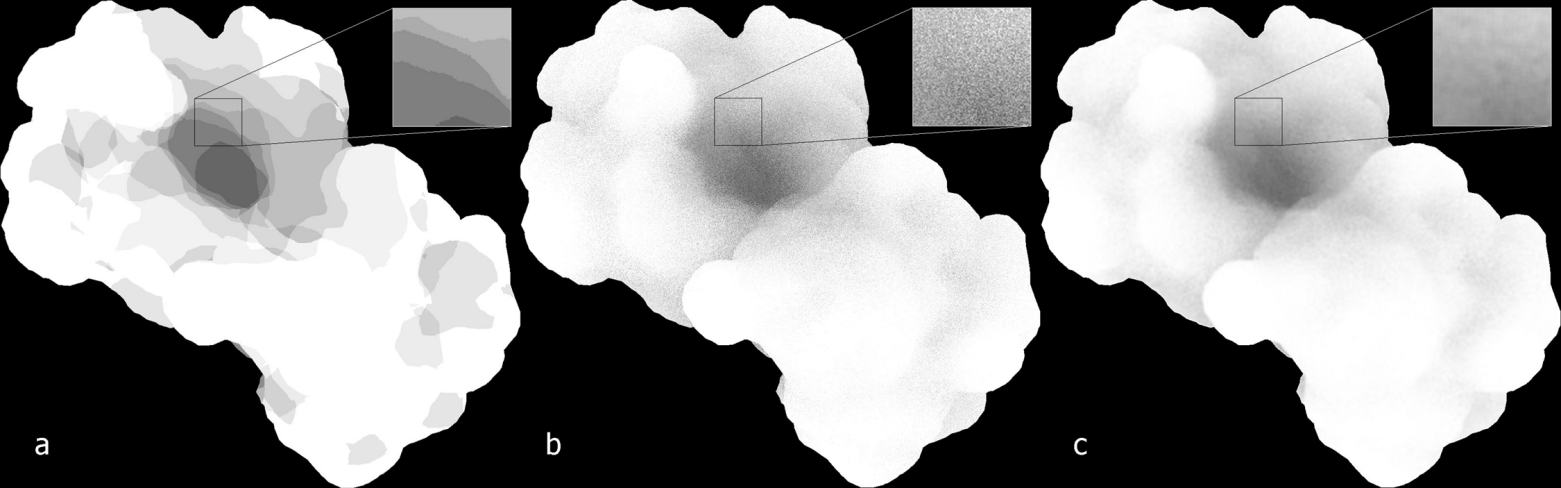
Point Sampling in SSAO. (a) AO with strong banding artifacts. (b) Randomized sampling. (c) Smoothing applied after the randomization.

The naïve method described up to here produces a strong banding artifact (Fig 6a). To eliminate this artifact, one can rotate the sampling sphere randomly for every pixel (Fig 6b). The random orientation of the sampling points inside the sphere eliminates the banding artifacts, but it introduces noise, so an additional smoothing step is required. The best trade-off between quality and speed for our purpose proved to be a 7 × 7 averaging kernel. This kernel can be decomposed into its two linear components along the x- and the y-axis, improving efficiency. Fig 6c shows the final result of the filtered point sampling.

### Line Sampling

The number of sampling points in the point-based method (Fig 5a and 5b) determines the quantization level of the AO coefficients. If the number is low, there will be noticeable granularity. To produce smooth variations between adjacent pixels one could simply increase the number of sample points. However, the quantization effects can be reduced more efficiently by using sampling lines orthogonal to the image plane inside the sampling sphere [23]. The intersection of each line with the surface is used to determine the fraction of the line that is hidden (Fig 5c). Adding up each line’s contribution to the sphere multiplied by its hidden fraction yields the final AO coefficient. Line sampling suppresses banding and granularity effects such that no randomization step is necessary. The hidden fraction of the line can be computed with a simple max-min calculation [23]. The simplicity of these calculations makes it even possible, at comparable cost, to consider a higher density of sampling lines compared to the above point sample density.

### Framebuffer Implementation

When AO is applied, the color and depth output are redirected into an offline framebuffer. (A framebuffer is a data structure that holds color and depth values of pixels.) For our purpose, we use a framebuffer with three attached color textures and one depth texture. The color image is stored in the first attached color texture, and depth is stored in the depth texture. For each pixel, a shader is invoked. (This is done by drawing two triangles that span the entire screen area; the rasterizer on the GPU will invoke a so-called fragment shader for each pixel covered.) This first shader takes the color and depth texture as input. The pixel is back-projected into 3D space, and the sampling points or lines are computed. The shader computes the ratio of hidden to visible samples and writes the output to the first color component of the second color texture. Additionally, two parameters are passed to this shader: the diameter of the sampling sphere and an intensity coefficient. The diameter is by default linked to the size of the displayed molecule, and thus needs to be user adjustable. A second shader then takes the second color texture as input. It blurs the coefficient in the x-direction by averaging. The result is stored in the first color component of the third color texture. The third and final shader takes the first and third color textures as input. It blurs the coefficient from the third color texture in the y-direction. Finally, it modulates the original colors with the blurred AO coefficients and writes the result into the screen framebuffer (a special framebuffer used for displaying contents on the screen).

These three steps are done for each single frame. The procedure is termed post-processing because it is conducted after the geometry of the scene has been rendered. Interactive frame rates can be maintained because all computations are done on the GPU. The CPU is required only for passing the name of the used shader to the GPU, for passing the name of the texture parameters, and for calling the functions that draw a rectangle that covers the screen.

## Results

Sculptor and its underlying libraries are implemented in C++. OpenGL is used for graphics rendering, and shader programs are written using Cg. The Cg run-time environment is linked to Sculptor and compiles all shader programs on program start. Compilation at run time has the advantage that it is guaranteed that the shader programs are compiled for the individual graphics card in the current computer. This means that whatever hardware is present, the Cg compiler will compile specifically for the latest feature set of the current machine, maximizing performance and compatibility. The software has been ported to current Windows, Macintosh, and Linux operating systems.

Taking AO into account when shading objects greatly enhances the appearance of 3D molecular structures (Figs 1 and 3). The figures also illustrate the adjustment of the important sampling diameter, which determines the scale of AO effect when working with biomolecules. In Fig 1c the diameter is relatively large, highlighting the molecular cavities. In Fig 1d, the diameter is decreased, providing a flatter appearance, but emphasizing molecular surface details on a finer level. Both depictions have their own individual advantages for the specific task at hand.

**Fig 7:**
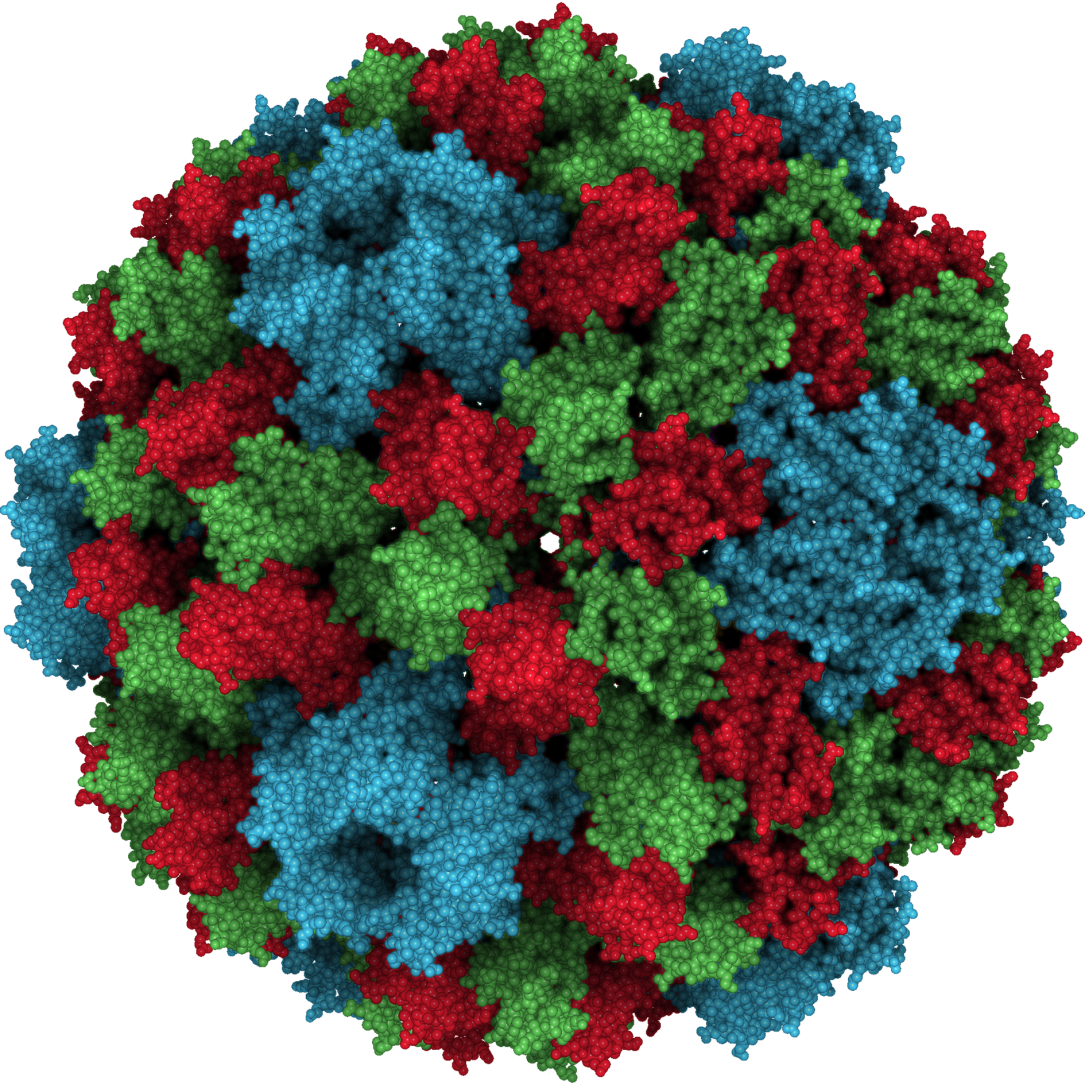
Three-way comparison of sampling size effect. Shown is a composite of CCMV images (PDB ID 1CWP) with three different heuristic sampling diameter settings. Adaption of the diameter highlights structural arrangements on different scales.

This multi-scale shading is particularly important high-resolution volumetric maps or for large atomic structures. In recent years, single-particle cryo-electron microscopy (cryo-EM) has advanced towards atomic resolution densities, and it is difficult to visualize features at multiple scales when rendering a 2.2 Å map at full detail (Fig 3). A larger sphere diameter is able to highlight tertiary structure features and the global molecular shape (Fig 3b), whereas a smaller sphere size can highlight secondary structure features (Fig 3c) or the fold of the polypeptide chain (Fig 3d). In a large atomic structure such as cowpea chlorotic mottle virus (CCMV) in Fig 7, the shading can be adjusted to provide different resolution depictions of the virus capsid surface. Adjusting the AO intensity level in conjunction with such sampling size variations can be useful for selecting the relative contrast of features. Both diameter and intensity levels can be updated interactively with sliders for this purpose.

To assess the impact of SSAO on graphics rendering speed, we conducted measurements on various hardware platforms (Section 2 in S1 Text). The performance penalty was negligible in all but the oldest systems we tested. We expect our SSAO implementation to impose no significant overhead on any current or future graphics systems. In terms of rendering quality, the line-based sampling is superior to the point-based one. Not only does it execute twice as fast at the same number of samples, it also delivers better looking results. Introducing noise to cope with banding artifacts is not necessary for line-based sampling, and thus results in a much smoother appearance of the SSAO effect. It also distributes contrasts better, with cavities receiving more and convexities less occlusion.

## Availability and Future Directions

In summary, we have presented an adaptation of SSAO to real-time molecular visualization that is uniquely useful for visualizing molecular features at multiple scales of interest. SSAO significantly enhances the perception of spatial features in large biomolecular structures. It works well in conjunction with biophysical data from various origins, resolutions, and with different rendering modes. Our performance tests showed that the computational cost of SSAO is negligible on modern platforms. Our Sculptor 2.1 builds are compatible with Linux, Macintosh, and Windows hardware from 2006 onward, making it a very resource-friendly graphics program. Sculptor is freely available under the LGPL license and it can be downloaded at http://sculptor.biomachina.org.

Several well-known molecular graphics packages are currently adopting global illumination schemes, although different approaches are taken by other groups:

The Python-based molecular viewer PMV [31] has supported conventional SSAO since 2011 for application in augmented reality and docking (Section 1 in S1 Text).

UCSF Chimera [32] will adopt AO lighting as part of its next-generation Chimera 2 program, which has not yet been released. The developers implemented “a direct shadowing approach that casts shadows from 100 to 200 directions. It packs the depth maps in a large 2D texture and a single shader pass computes all the shadows. On mid-range graphics it can display large atomic structures such as a ribosome at interactive frame rate” (Tom Goddard, pers. comm.). Direct shadows require re-computation of shadow maps whenever the model changes, while SSAO doesn’t have that overhead. The Chimera developers believe that in their applications most molecular scenes are rather static and so the direct shadow approach is viable. SSAO is more attractive when the scene is very dynamic, such as in rendering of molecular dynamics trajectories within Sculptor.

VMD [33, 34] recently adopted a “fully interactive GPU-accelerated ray tracing, which allows traditional shadows, AO lighting, reflections, depth-of-field focal blur, at frame rates ranging from 10 frames per second on older GPUs, up to 30 frames per second or more on the latest NVIDIA gaming GPUs” (John Stone, pers. comm.). The VMD GPU ray tracer started out as a way of getting high-quality movie renderings done more quickly on large supercomputers [34]. The ray tracing feature has recently been released in VMD 1.9.2 for the 64-bit Linux build.

Despite the sophistication of direct shading and ray tracing, working in screen space has some inherent advantages. First, it is completely independent of the complexity of the geometry of the scene. AO is computed on a per pixel, not a per vertex basis. This makes SSAO extremely efficient (Section 2 in S1 Text). Although our applications are mostly performed on workstations, we note that SSAO is applicable for interactive rendering on low-power platforms such as mobile phones, tablets, and web browsers.

The most important practical advantage of our SSAO implementation (besides speed) is the user-adjustable sphere size parameter. This feature is particularly beneficial for multi-scale docking studies, because it selectively highlights specific size cavities and binding pockets through improved lighting. The user selection of sampling size could become a potential issue if one wanted to make movies that zoom in/out over a wide spatial scale while retaining a fixed sampling sphere diameter. In such simulations the zooming might lead to visible granularity artifacts when the depth-buffer resolution becomes very low relative to the projected sample spheres. One could compensate for such limitations by additional oversampling in the line-based approach, by adapting the sample sphere diameter automatically, or by disabling the SSAO effect as needed.

Another minor limitation of SSAO is that it does not capture the shadowing contributions from objects that are off-screen. When displaying “whole structure” views of molecular assemblies such as the ones selected in this paper, the lack of influence from off-screen geometry is not a problem, but it can become an issue when zooming in on an area of structure, where some of the structure is clipped by the view frustum, and it can be distracting when making movies. This limitation could be addressed in future work by the multi-layer depth peeling SSAO [28], but at additional cost in rendering performance.

Sculptor offers many different modes of structural representation of biomolecules and 3D density maps, such as van der Waals, stick, ribbon, isosurface, and volume rendering. Some Sculptor techniques are designed to speed up the rendering process for dynamic scenes using customized vertex shaders. Others conduct high-quality per pixel lighting, or non-lighting effects such as depth cueing. Our SSAO implementation is fully integrated with these rendering techniques due to the post-processing design. It would be possible in future work to make slight modifications to the rendering pipeline, which would allow more data to be available in the post-processing. This is exploited in [35], where directional information is used to improve occlusion and to add simple reflections.

Another promising opportunity would be to combine the approach used by [16] (GLSL splatting or synthetic density map schemes) for object-space AO, with the selective probe radius scheme used in this paper. The use of an object-space sampling sphere would enable a more precise rendering of constant scale spatial features in molecular systems. In screen space we provide a qualitative, user-adjustable parameter, but it would be desirable to map that diameter to object space and to investigate quantitatively what “default” sphere size is useful in a wide range of biomolecular applications. For example, in cryo-EM density maps, beta sheets are optimally visible at about 5 Å resolution and alpha helices at 8 Å resolution. This might produce a new molecular graphics approach that combines the benefits of both methods, and the multi-scale rendering is somewhat orthogonal to the direction taken by other global illumination schemes such as ray tracing.

## Supporting Information

S1 Text A review of online technical literature and SSAO performance tests. (PDF)
