# Supplementary material for: Multi-scale Visualization of Molecular Architecture Using Real-Time Ambient Occlusion in Sculptor
Source: PLoS Comput Biol. 2015 Oct 27;11(10):e1004516. doi: 10.1371/journal.pcbi.1004516 (PMC4623981; doi:10.1371/journal.pcbi.1004516)
Supplement: S1 Text — (PDF) [file pcbi.1004516.s001.pdf]

Supplementary Material for "Multi-Scale Visualization of Molecular Architecture Using Real-Time Ambient Occlusion in Sculptor"

Manuel Wahle and Willy Wriggers

September 28, 2015

1 Online Technical Literature

In the main text we cited publications in printed journals or proceedings. However, given the technical nature of the work, much recent progress has been published online. We recommend that interested readers should extend their literature search to the following web sites for a more comprehensive view of prior work:

- [1] is the molecular graphics documentation of the CCP4 suite of programs, with information specific to lighting, shadows, and shaders.
- [2] describes various 3D navigation and modeling tools for cellular biology by the Life Explorer Initiative.
- [3] describes the chemlab.graphics package. The purpose of the package is to provide a solid library to develop 3D applications to display chemical data in a flexible way.
- [4] shows usage examples of the open-source general packing algorithm autoPACK running in the molecular graphics package PMV.
- [5] shows an SSAO-based application of PMV in augmented reality.
- [6] highlights UnityMol, which uses the Unity3D game engine to create a molecular viewer and serious games.
- [7–9] highlight applications of Cyril Crassin's voxel cone AO (see main text) by NVIDIA Corporation.

We thank the anonymous reviewers for suggesting some of these sites.

2 Performance Tests

To assess the impact of our implementation of SSAO on rendering speed, we conducted tests on various hardware platforms. The performance of GPUs and CPUs varies widely among computers in the field today, since computers are replaced less frequently now than they were a decade ago. We first tested the performance on an older, low-end system (Athlon X2 / GeForce 6600LE) to ensure backward compatibility for a wide range of users.

Table 1 shows how computing time scales directly with the number of pixels covered. With the line-based method, interactive frame rates on the low end system are guaranteed even with a model that covers the entire screen area. For practical purposes, on standard definition displays and older graphics cards one can assume that 0.5 million is an upper bound on the number of pixels covered by the molecule. (Models are typically not displayed full screen, and controls and window with settings are opened next to the model.)

Table 1: **Frame time (in milliseconds) for SSAO sampling methods.**

# occupied pixel	none	point-based	line-based
0.25 million	1	51	26
0.5 million	2	109	50
0.75 million	3	170	73
1.0 million	4	230	97

Tests were performed on an Athlon X2 / GeForce 6600LE system.

Next, we tested the method on a classic mid-range system (Core 2 Duo 3GHz / GeForce8800GTX) that was connected to two projectors for stereoscopic rendering at full screen (0.79 million pixels per projector). Intel's Core 2 Duo chips were released way back in 2006, but they still perform well today in video game applications [10]. The point-based method required only 6 milliseconds compute time, and the line-based method only 2 milliseconds. Since two images for each frame were computed, these values must be doubled. This allowed for about 80 frames per second with the point-based sampling, and up to about 250 frames per second with the line-based method using stereoscopic rendering.

On a more recent mid-range system with a GeForce GTX295 graphics card, the impact on rendering speed was no longer measurable, even on a high-definition screen with fully occupied 1.9 million pixels.

In summary, the performance penalty was negligible in all but the oldest systems we tested. We expect our implementation to impose no significant overhead on any current or future graphics systems.

References

1. Collaborative Computational Project No. 4. CCP4 Molecular Graphics Documentation - Lighting, Shadows and Shaders. http://www.ccp4.ac.uk/MG/ccp4mg_help/lighting.html

2. Life Explorer Initiative. 3D Navigation and Modeling Tools for Cellular Biology. http://www.lifeexplorer.info/

3. Chemlab Library. Graphics and Visualization Documentation. http://chemlab.readthedocs.org/en/latest/graphics.html

4. autoPACK Packing Algorithm. Experiments and Image Gallery. http://www.autopack.org/gallery

5. Edgaras Art - YouTube. Augmented Reality of Flexible DNA with Ambient Occlusion (SSAO). https://www.youtube.com/watch?v=_OYTYIwC5g0

6. Baaden M. UnityMol. http://www.baaden.ibpc.fr/umol/

7. NVIDIA Corporation. Interactive Indirect Illumination Using Voxel Cone Tracing. http://tinyurl.com/lzganhh

8. NVIDIA Corporation. NVIDIA VXGI Voxel Cone Tracing. https://developer.nvidia.com/vxgi

9. Panteleev A. Practical Real-Time Voxel-Based Global Illumination for Current GPUs. http://tinyurl.com/m3q6hdj

10. Chacos B. Why Moore's Law, not mobility, is killing the PC. PC World. 2013. http://www.pcworld.com/article/2030005
